# Reduced reactive hyperemia of the brachial artery in diabetic patients assessed by repeated measurements: The FMD‐J B study

**DOI:** 10.14814/phy2.15786

**Published:** 2023-08-22

**Authors:** Nobuyuki Masaki, Takeshi Adachi, Hirofumi Tomiyama, Takahide Kohro, Toru Suzuki, Tomoko Ishizu, Shinichiro Ueda, Tsutomu Yamazaki, Tomoo Furumoto, Kazuomi Kario, Teruo Inoue, Shinji Koba, Yasuhiko Takemoto, Takuzo Hano, Masataka Sata, Yutaka Ishibashi, Koichi Node, Koji Maemura, Yusuke Ohya, Taiji Furukawa, Hiroshi Ito, Yukihito Higashi, Akira Yamashina, Bonpei Takase

**Affiliations:** ^1^ Department of Intensive Care Medicine National Defense Medical College Tokorozawa Japan; ^2^ Department of Cardiology National Defense Medical College Tokorozawa Japan; ^3^ Department of Cardiology Tokyo Medical University Tokyo Japan; ^4^ Department of Hospital Planning and Management, Medical Informatics Jichi Medical University School of Medicine Tochigi Japan; ^5^ Cardiovascular Medicine University of Leicester Leicester UK; ^6^ Cardiovascular Division Institute of Clinical Medicine, University of Tsukuba Ibaraki Japan; ^7^ Department of Clinical Pharmacology and Therapeutics University of the Ryukyu School of Medicine Okinawa Japan; ^8^ Department of Clinical Epidemiology and Systems, Faculty of Medicine The University of Tokyo Tokyo Japan; ^9^ Department of Cardiovascular Medicine Hokkaido University Graduate School of Medicine Sapporo Japan; ^10^ Division of Cardiovascular Medicine Jichi Medical University School of Medicine Tochigi Japan; ^11^ Dokkyo Medical University; Nasu Red Cross Hospital Tochigi Japan; ^12^ Department of Medicine, Division of Cardiology Showa University School of Medicine Tokyo Japan; ^13^ Department of Internal Medicine and Cardiology Osaka City University Graduate School of Medicine Osaka Japan; ^14^ Department of Medical Education and Population‐based Medicine, Postgraduate School of Medicine Wakayama Medical University Wakayama Japan; ^15^ Department of Cardiovascular Medicine Institute of Health Biosciences, The University of Tokushima Graduate School Tokushima Japan; ^16^ Department of General Medicine Shimane University Faculty of Medicine Shimane Japan; ^17^ Department of Cardiovascular Medicine Saga University Saga Japan; ^18^ Department of Cardiovascular Medicine, Course of Medical and Dental Sciences, Graduate School of Biomedical Sciences Nagasaki University Nagasaki Japan; ^19^ The Third Department of Internal Medicine University of the Ryukyus Okinawa Japan; ^20^ Department of Internal Medicine Teikyo University School of Medicine Tokyo Japan; ^21^ Department of Cardiovascular Medicine Okayama University Graduate School of Medicine, Dentistry, and Pharmaceutical Sciences Okayama Japan; ^22^ Department of Regenerative Medicine Research Institute for Radiation Biology and Medicine, Hiroshima University Hiroshima Japan

**Keywords:** clinical study, diabetes mellitus, endothelial function, hyperglycemia, reactive hyperemia

## Abstract

Type 2 diabetes mellitus (T2DM) is a major cause of microvascular dysfunction. However, its effect on blood flow patterns during ischemic demand has not been adequately elucidated. In this study, we investigated the hypothesis that microvascular dysfunction in patients with T2DM manifests as brachial reactive hyperemia (BRH), defined as the ratio of peak blood flow velocities in a brachial artery before and after forearm cuff occlusion. The study enrolled 943 subjects (men, *n* = 152 [T2DM] and *n* = 371 [non‐T2DM]; women, *n* = 107 [T2DM] and *n* = 313 [non‐T2DM], respectively) with no history of cardiovascular disease. Semiautomatic measurements were obtained three times at 1.5‐year intervals to confirm the reproducibility of factors involved in BRH for each sex. An age‐adjusted mixed model demonstrated attenuated BRH in the presence of T2DM in both men (*p* = 0.022) and women (*p* = 0.031) throughout the study period. Post hoc analysis showed that the estimated BRH was significantly attenuated in patients with T2DM regardless of sex, except at baseline in women. In multivariate regression analysis, T2DM was a negative predictor of BRH at every measurement in men. For women, BRH was more strongly associated with alcohol consumption. Repeated measurements analysis revealed that T2DM was associated with attenuated postocclusion reactive hyperemia.

## 1 INTRODUCTION

Vascular endothelial dysfunction has been shown to have a significant impact on the development of cardiovascular disease and has been the subject of numerous studies (Celermajer et al., 1992; Deanfield et al., 2007; Matsuzawa et al., 2013; Takase et al., 1998). The most widely used test of endothelial function is flow‐mediated dilation (FMD). The FMD test involves temporarily blocking blood flow and then observing dilation of the vessels over time after restoration of blood flow. Reactive hyperemia in the brachial artery can also be measured by Doppler ultrasound. Compared with FMD, the magnitude of reactive hyperemia better reflects the degree of peripheral endothelial function (Philpott & Anderson, 2007; Tagawa et al., 1994). Several previous studies have reported on the usefulness of the indices of reactive hyperemia for predicting adverse cardiovascular outcomes (Anderson et al., 2011; Calderaro et al., 2008; Huang et al., 2007; London et al., 2004).

Indices of reactive hyperemia in the brachial arteries are reported to be related to blood glucose levels (Mitchell et al., 2004), insulin resistance caused by short‐term physical inactivity (Hamburg et al., 2007), number of components of the metabolic syndrome present (Hamburg et al., 2008), and past history of cardiovascular disease (Hamburg et al., 2011). Type 2 diabetes mellitus (T2DM) is a major cause of microvascular dysfunction and leads to diabetic angiopathy of the retinal, renal, cardiac, and cerebral arteries as well as arteries in the lower extremities (Crasto et al., 2021). However, there is limited evidence to suggest that T2DM has an impact on reactive hyperemia in the brachial arteries (Keymel et al., 2011).

There are several possible reasons why reactive hyperemia in the brachial arteries has not been investigated in patients with T2DM. First, reactive hyperemia is influenced by many background characteristics, including age, sex, body habitus, and concomitant diseases, and the effect of T2DM may not be detectable in a single evaluation. Second, more advanced technical training is required in order to obtain accurate measurements (Rosenberry & Nelson, 2020). The testing procedure also affects measurements of brachial blood flow. For example, hyperemic flow increases with cuff occlusion time. Induction of reactive hyperemia depends on the magnitude of tissue ischemia caused by cuff occlusion, which is attenuated in elderly subjects (Rosenberry et al., 2019). Third, many analytical approaches can be used for quantifying reactive hyperemia, such as velocity and flow rate, and measurements may be obtained at various time points in the cardiac cycle, making it difficult to comparisons between studies.

Therefore, in this study, we tested the hypothesis that microvascular dysfunction in patients with T2DM manifests in a specific blood flow pattern in the brachial arteries after occlusive ischemia using a standardized method. We have established a multicenter registry known as the FMD‐J, in which all data were obtained using a semiautomated FMD instrument (Tomiyama et al., 2012). This device can obtain more accurate blood flow and velocity profiles by adjusting the angle of incidence and velocity gradient from the center to the edge across the lumen (O'Rourke & Nichols, 2004). Semiautomatic measurements were obtained at time points spaced 1.5 years apart with collection of laboratory data and lifestyle information at the same time points (Tomiyama et al., 2012). This allowed us to confirm the reproducibility of the results obtained. The ratio of peak blood flow velocity after cuff release to that of the baseline value was used to quantify brachial reactive hyperemia (BRH) instead of absolute velocity values. This ratio also minimizes the influence of angle of incidence and individual differences in resting blood flow velocity. The objective measurement method used, the parameters measured, and repeated measurements obtained will clarify the effect of T2DM on reactive hyperemia.

## 2 METHODS

### 2.1 Participants

The study involved patients who had participated in the Flow‐Mediated Dilation Japan (FMD‐J) B study (Tomiyama et al., 2012). The FMD‐J was a prospective multicenter study conducted at 22 university hospitals and affiliated clinics in Japan that examined the usefulness of FMD assessment in the management of patients at risk of cardiovascular disease. Detailed information on the FMD‐J study protocol and its participants is publicly available (Tomiyama et al., 2012). The FMD‐J B study was intended to elucidate the effect of lifestyle‐related diseases on vascular function and enrolled patients aged 20–74 years with no history of cardiovascular disease who had had at least one cardiovascular risk factor (e.g., hypertension or diabetes), for at least 6 months.

### 2.2 Study protocol

FMD and BRH were measured at 1.5‐year intervals for 3 years (i.e., baseline, 1.5 years, and 3 years). Treatment for disease, medications received, lifestyle factors, and laboratory values were assessed at each time point.

Patients were defined as having hypertension and hyperlipidemia if they were receiving medications for these conditions during the study period. T2DM was defined according to the American Diabetes Association guideline (American Diabetes Association Professional Practice Committee, 2022) as being on treatment for diabetes or having a fasting blood glucose level of >126 mg/dL or a glycated hemoglobin (HbA_1c_) of >6.5% recorded during the observation period.

Data for men and women were analyzed separately. The cross‐sectional BRH data were assessed at each of the three time points.

### 2.3 Measurement protocol

FMD was measured using a semiautomated ultrasound instrument (UNEXEF18G; UNEX Co., Nagoya, Japan), which is used specifically for measurement of FMD. All sonographers at the participating institutions received training on the use of a standardized protocol for FMD measurement and on scanning and analysis of the recordings in a central laboratory located at Tokyo Medical University. All brachial artery scans obtained during measurement of FMD were sent from the participating institutions to the central laboratory by USB flash drive and were analyzed individually by an experienced reader who was blinded to all patient information.

The study protocol was approved by the ethics committees of the participating institutions and performed in accordance with the Good Clinical Practice guidelines. Informed consent was obtained from all participants.

Participants fasted from the night before FMD examination and abstained from alcohol, smoking, caffeine, and antioxidant vitamins on the day of the examination. The participants were kept in the supine position in a quiet, dark, and air‐conditioned room (maintained at a constant temperature of 23°C–26°C) throughout the study. A 23‐gauge polyethylene catheter was inserted into the left deep antecubital vein to obtain blood samples. Blood pressure was measured in the left arm using a mercury sphygmomanometer with an appropriately sized cuff and recorded to the nearest 2 mm Hg. Pulse pressure was calculated as systolic blood pressure minus diastolic blood pressure.

The cuff was placed on the forearm and the ultrasound probe was positioned proximally after the subject had been in the supine position for at least 20 min. The cuff pressure was standardized to a pressure of 50 mm Hg higher than the systolic pressure measured before the test (Tomiyama et al., 2012) in conformity with the current recommendations (Thijssen et al., 2011, 2019). The cuff inflation time was 5 min. After deflation, blood flow velocity and vessel lumen diameter were monitored continuously. Vessel lumen diameter was measured in the end‐diastolic phase of the cardiac cycle. All examiners were blinded to the patients' clinical information. The intraclass correlation coefficient between each participating institution and the central laboratory has been reported previously (Tomiyama et al., 2015).

Blood flow velocity values were calculated by Doppler ultrasound as follows. Sampling positions were automatically set at equal intervals along the diameter of the arterial lumen. At each sampling location, the time variation in blood flow velocity measured at a sampling frequency higher than 500 Hz was plotted. Finally, cross‐sectional averages were calculated for each sampling location along the diameter of the arterial lumen, assuming that the concentric velocities were the same. BRH was defined as the increase in blood flow velocity expressed as the ratio of the highest cross‐sectional average velocity to its baseline value.

### 2.4 Statistical analysis

Data are expressed as the mean ± standard deviation or as the mean ± standard error for longitudinal analysis. The data were tested for normality using the Shapiro–Wilk test. Variables with a non‐normal distribution are presented as the median and interquartile range. Categorical variables were compared using the chi‐squared test or Fisher's exact test as appropriate. Correlations between two continuous variables were determined by Pearson's method. We also compared fitting models, including linear, multiplier, and exponential models, and applied the best‐fitted curve.

The analyses were performed separately for each sex. A mixed model was used to determine whether BRH differed in repeated measures between patients with T2DM and those without T2DM. In the analysis, T2DM was included as a main effect and age was adjusted for as a covariate. The facility where the examination was performed was included as a random effect. Univariate and multivariate linear regression analyses were performed to identify factors contributing to BRH and to elucidate the significance of T2DM. The multivariate stepwise models included factors identified to be potentially associated with BRH in univariate analysis (*p* < 0.1). Age was fixed as an independent variable. HbA_1c_ was excluded from the multivariate regression analysis owing to a high number of missing values. All statistical analyses were performed using SPSS version 22.0 (SPSS Japan, Tokyo, Japan). All tests were two‐tailed, and a *p* value <0.05 was considered statistically significant.

## 3 RESULTS

### 3.1 Clinical characteristics at baseline

**FIGURE 1:**
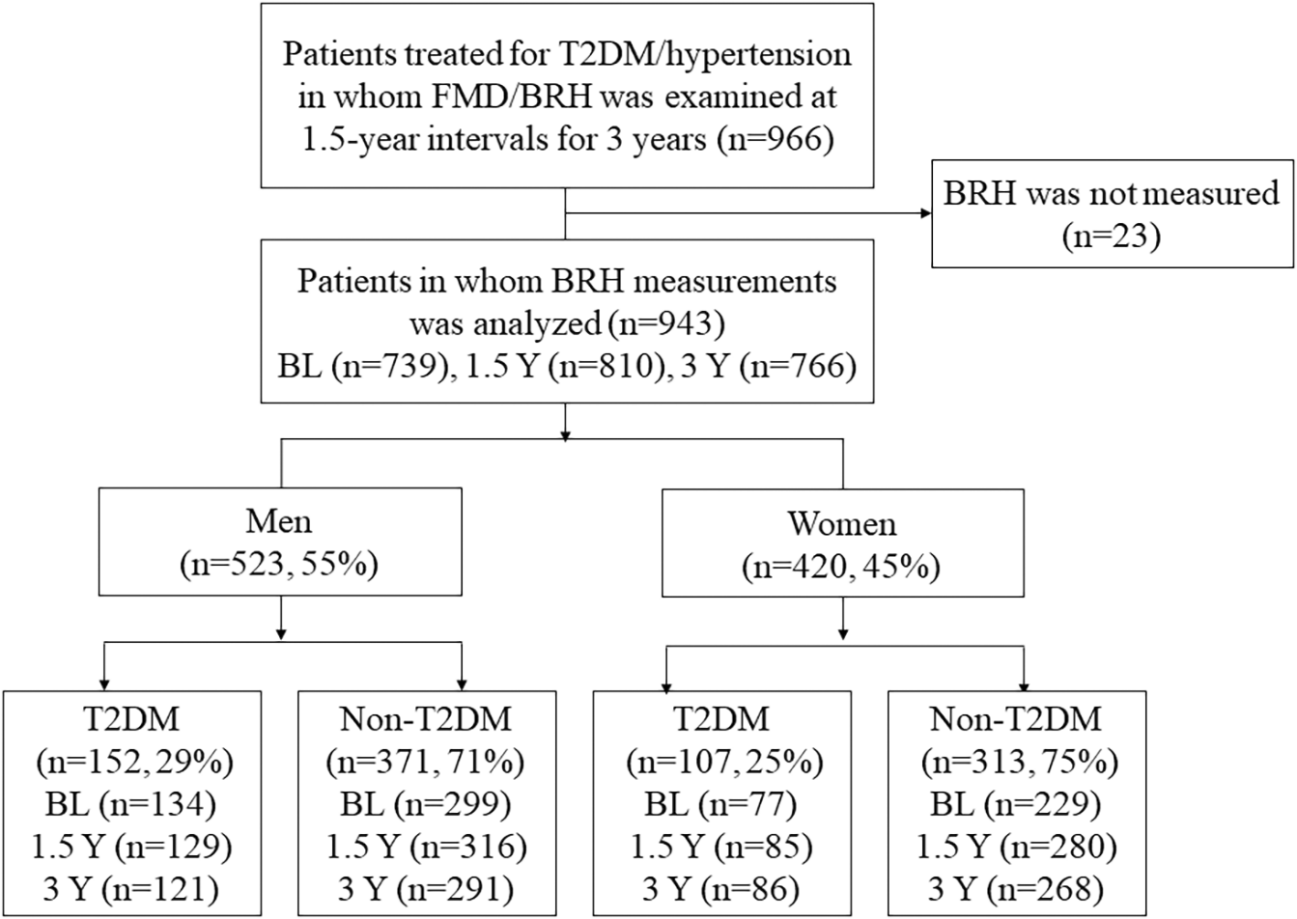
Flowchart of study. A total of 966 patients were enrolled from 22 centers. Data were analyzed for 943 patients who were evaluated for brachial hyperemia at one time point at least. The actual number of patients who underwent BRH testing at each time point is shown. BL, baseline; BRH, brachial reactive hyperemia; FMD, flow‐mediated dilation; T2DM, type 2 diabetes mellitus; Y, years.

A total of 966 patients were enrolled in the FMD‐J B study (Figure 1). We analyzed the data for 943 patients in whom BRH was tested at least once, including 573 patients (61%) who underwent BRH examinations on all occasions. We classified 259 patients (27%) into the T2DM group based on medication history and laboratory data. Figure 1 shows the actual numbers of patients who underwent BRH testing because the composition of patients in whom the test was performed varied over time. Table 1 shows the differences in clinical features between the T2DM group and the non‐T2DM group. Subjects in the T2DM group were more likely to be older, to have hyperlipidemia, to have a higher body mass index (BMI), and to be on a statin. Total cholesterol levels were lower in patients with T2DM because of antilipidemic treatment. Alcohol consumption was less common in women than in men and more common in subjects without T2DM than in those with T2DM.

**TABLE 1:** Demographic and clinical characteristics.

	Men		*p* value	Women		*p* value
	T2DM	Non‐T2DM		T2DM	Non‐T2DM	
	(*n* = 152)	(*n* = 371)		(*n* = 107)	(*n* = 313)	
Age (years)	63 ± 9	60 ± 11	**0.008**	65 ± 8	62 ± 9	**0.002**
BMI (kg/m^2^)	26 ± 4	25 ± 3	**0.002**	25 ± 4	24 ± 4	**0.002**
Hypertension, *n* (%)	144 (95)	361 (97)	0.144	103 (96)	303 (97)	0.787
Hyperlipidemia, *n* (%)	73 (48)	126 (34)	**0.003**	62 (58)	133 (42)	**0.006**
Current smoking, *n* (%)	28 (18)	62 (17)	0.638	7 (7)	21 (7)	0.952
Past smoking, *n* (%)	78 (51)	187 (50)	0.850	11 (10)	30 (10)	0.834
Alcohol consumption, *n* (%)	93 (61)	238 (64)	0.523	15 (14)	74 (24)	**0.035**
Regular exercise, *n* (%)	77 (51)	206 (56)	0.310	59 (55)	175 (56)	0.890
Calcium channel blocker, *n* (%)	104 (68)	240 (65)	0.414	70 (65)	178 (57)	0.120
ARB/ACE inhibitor, *n* (%)	111 (73)	251 (68)	0.227	69 (64)	181 (58)	0.226
Beta‐blocker, *n* (%)	36 (24)	56 (15)	**0.019**	20 (19)	56 (18)	0.853
Diuretic therapy, *n* (%)	45 (30)	101 (27)	0.581	33 (31)	71 (23)	0.091
Aldosterone antagonist, *n* (%)	5 (3)	7 (2)	0.331	5 (5)	10 (3)	0.477
Statin therapy, *n* (%)	57 (38)	81 (22)	**<0.001**	49 (46)	89 (28)	**0.001**
Fibrate therapy, *n*, (%)	6 (4)	11 (3)	0.565	0 (0)	3 (1)	0.574
Oral antidiabetic therapy, *n* (%)	55 (36)	0 (0)	**<0.001**	39 (36)	0 (0)	**<0.001**
Insulin, *n* (%)	1 (1)	0 (0)	0.291	3 (3)	0 (0)	**0.016**
Aspirin, *n* (%)	27 (18)	57 (15)	0.497	14 (13)	32 (10)	0.413
Total cholesterol (mg/dL)	187 ± 31	197 ± 32	**0.003**	201 ± 34	210 ± 30	**0.009**
HDL cholesterol (mg/dL)	53 ± 15	55 ± 15	0.214	60 ± 16	65 ± 15	**0.001**
Triglycerides (mg/dL)	148 ± 84	146 ± 98	0.824	132 ± 60	107 ± 52	**<0.001**
Glucose (mg/dL)	124 ± 25	100 ± 9	**<0.001**	126 ± 27	96 ± 8	**<0.001**
Creatinine (mg/dL)	0.87 ± 0.21	0.85 ± 0.19	0.323	0.64 ± 0.18	0.63 ± 0.14	0.379
eGFR (mL/min)	73 ± 17	75 ± 18	0.173	76 ± 22	76 ± 17	0.709
Uric acid (mg/dL)	6.18 ± 1.26	6.28 ± 1.30	0.431	5.31 ± 1.06	4.92 ± 1.13	**0.002**
HbA_1c_ (%)^a^	6.50 ± 0.86	5.65 ± 0.31	**<0.001**	6.72 ± 0.69	5.68 ± 0.31	**<0.001**
Alcohol intake (g/day)^b^	31 ± 21	35 ± 23	0.243	18 ± 17	24 ± 22	0.397
Number of cigarettes (/day)^c^	17 ± 6	16 ± 8	0.646	13 ± 6	12 ± 7	0.704

Abbreviations: ACE, angiotensin‐converting enzyme; ARB, angiotensin receptor blocker; BMI, body mass index; eGFR, estimated glomerular filtration rate; HbA_1c_, glycated hemoglobin; HDL, high‐density lipoprotein; T2DM, type 2 diabetes mellitus.

*Note*: The data are shown as the mean ± standard deviation. *p* values <0.05 are shown in bold. The data for BMI, smoking status, alcohol consumption and exercise habits, medication, and laboratory results expressed in the table were those obtained at baseline. The data for hypertension, hyperlipidemia, and T2DM were obtained over the 3‐year study period.

^a^ *n* = 129, *n* = 288, *n* = 98, and *n* = 249 for men with T2DM, men without T2DM, women with T2DM, and women without T2DM, respectively.

^b^ *n* = 93, *n* = 238, *n* = 15, and *n* = 74 for male drinkers with T2DM, male drinkers without T2DM, female drinkers with T2DM, and female drinkers without T2DM, respectively.

^c^ *n* = 28, *n* = 62, *n* = 7, and *n* = 21 for male smokers with T2DM, male smokers without T2DM, female smokers with T2DM, and female smokers without T2DM, respectively.

### 3.2 Comparison of BRH between subjects with and without T2DM

Table 2 shows the FMD and BRH data at each of the three time points. BRH was significantly attenuated in subjects with T2DM compared with those without T2DM at baseline, 1.5 years, and 3 years. The cross‐sectional data show that systolic blood pressure at the time of examination tended to be higher and diastolic blood pressure tended to be lower in subjects with T2DM than in those without T2DM. Pulse pressure was higher in subjects with T2DM. BRH was lower in subjects with T2DM at all time points regardless of sex, except at baseline in women.

**TABLE 2:** Results of FMD and BRH examinations.

	Men		*p* value	Women		*p* value
	T2DM	Non‐T2DM		T2DM	Non‐T2DM	
Patients, *n*	152	371		107	313	
Diameter at baseline (mm) BL	4.38 ± 0.56	4.46 ± 0.53	0.097	3.76 ± 0.55	3.64 ± 0.53	**0.043**
Diameter at baseline (mm) 1.5 Y^a^	4.42 ± 0.52	4.45 ± 0.52	0.575	3.76 ± 0.56	3.68 ± 0.53	0.192
Diameter at baseline (mm) 3 Y^b^	4.42 ± 0.53	4.44 ± 0.55	0.765	3.74 ± 0.54	3.68 ± 0.52	0.328
FMD (%) BL	5.22 ± 2.76	4.45 ± 2.49	**0.002**	4.33 ± 2.69	5.49 ± 3.03	**0.001**
FMD (%) 1.5 Y^a^	4.83 ± 2.75	4.72 ± 2.97	0.711	4.79 ± 2.78	5.41 ± 3.17	0.097
FMD (%) 3 Y^b^	5.11 ± 4.04	4.77 ± 3.47	0.384	4.85 ± 2.84	5.32 ± 3.22	0.215
BRH BL^c^	3.25 ± 1.88	4.19 ± 2.38	**<0.001**	3.06 ± 1.83	3.47 ± 2.03	0.123
BRH 1.5 Y^d^	3.77 ± 2.07	4.75 ± 2.95	**0.001**	3.33 ± 1.98	4.04 ± 2.26	**0.009**
BRH 3 Y^e^	3.92 ± 1.90	4.78 ± 2.65	**0.001**	3.34 ± 2.01	3.92 ± 2.08	**0.024**
Heart rate (bpm) BL	66 ± 11	63 ± 9	**0.006**	67 ± 13	66 ± 10	0.526
Heart rate (bpm) 1.5 Y^a^	65 ± 12	63 ± 10	**0.016**	66 ± 12	65 ± 11	0.404
Heart rate (bpm) 3 Y^b^	66 ± 12	62 ± 10	**<0.001**	66 ± 12	64 ± 10	0.250
Systolic BP (mmHg) BL	132 ± 14	130 ± 14	0.108	135 ± 18	131 ± 18	0.101
Systolic BP (mmHg) 1.5 Y^a^	134 ± 17	129 ± 14	**0.004**	134 ± 17	130 ± 17	0.077
Systolic BP (mmHg) 3 Y^b^	132 ± 17	130 ± 15	0.216	131 ± 17	128 ± 16	0.137
Diastolic BP (mmHg) BL	80 ± 9	82 ± 9	0.081	76 ± 11	80 ± 10	**0.001**
Diastolic BP (mmHg) 1.5 Y^a^	79 ± 11	80 ± 10	0.501	75 ± 10	78 ± 10	**0.022**
Diastolic BP (mmHg) 3 Y^b^	77 ± 11	79 ± 11	0.064	73 ± 10	77 ± 10	**0.003**
Pulse pressure (mmHg) BL	52 ± 12	48 ± 12	**0.001**	59 ± 16	52 ± 15	**<0.001**
Pulse pressure (mmHg) 1.5 Y^a^	54 ± 14	49 ± 12	**<0.001**	58 ± 14	52 ± 14	**<0.001**
Pulse pressure (mmHg) 3 Y^b^	54 ± 14	50 ± 12	**0.001**	57 ± 14	51 ± 12	**<0.001**

Abbreviations: BL, baseline; BP, blood pressure; BRH, brachial reactive hyperemia; FMD, flow‐mediated dilation; T2DM, type 2 diabetes mellitus; Y, years.

*Note*: The data are shown as the mean ± standard deviation. *p* values <0.05 are shown in bold.

^a^ *n* = 131, *n* = 328, *n* = 91, and *n* = 288 for men with T2DM, men without T2DM, women with T2DM, and women without T2DM, respectively.

^b^ *n* = 124, *n* = 293, *n* = 90, and *n* = 271 for men with T2DM, men without T2DM, women with T2DM, and women without T2DM, respectively.

^c^ *n* = 134, *n* = 299, *n* = 77, and *n* = 229 for men with T2DM, men without T2DM, women with T2DM, and women without T2DM, respectively.

^d^ *n* = 129, *n* = 316, *n* = 85, and *n* = 280 for men with T2DM, men without T2DM, women with T2DM, and women without T2DM, respectively.

^e^ *n* = 121, *n* = 291, *n* = 86, and *n* = 268 for men with T2DM, men without T2DM, women with T2DM, and women without T2DM, respectively.

### 3.3 Repeated measurements of BRH

**FIGURE 2:**
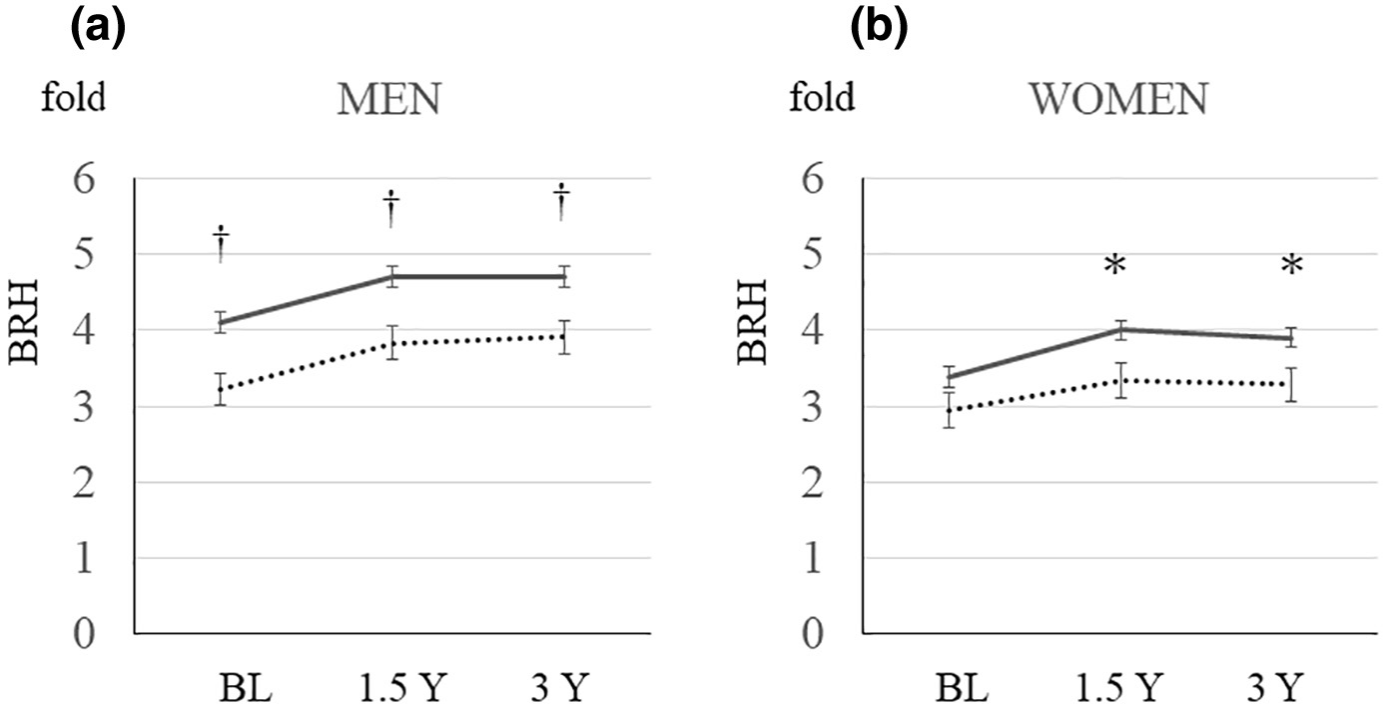
Age‐adjusted BRH according to T2DM status. The graph shows the time course of BRH measurements adjusted for age (A, men; B, women). With the except of BRH at baseline in women, BRH was significantly lower in the T2DM group than in the non‐T2DM group. The data are shown as the mean ± standard error. The solid lines are for the non‐T2DM group, and the dotted lines are for the T2DM group. *p < 0.05, T2DM versus non‐T2DM, ^†^ *p* < 0.01, T2DM versus non‐T2DM. BL, baseline; BRH, brachial reactive hyperemia; T2DM, type 2 diabetes mellitus; Y, years.

Figure 2 shows the time course of changes in BRH for each sex and adjusted for age. In the mixed model, repeated measurements demonstrated that BRH was lower in subjects with T2DM whether male (*n* = 523, *p* = 0.022) or female (*n* = 420, *p* = 0.031). There was no interaction effect between measurement time points and T2DM or BRH. The age‐adjusted estimated BRH was lower in men with T2DM than in men without T2DM (mean ± standard error 3.22 ± 0.21 [T2DM] vs. 4.11 ± 0.14 [non‐T2DM], *p* < 0.001 at baseline; 3.84 ± 0.21 vs. 4.71 ± 0.14, *p* = 0.001 at 1.5 years; and 3.91 ± 0.22 vs. 4.70 ± 0.14, *p* = 0.003 at 3 years) and in women with the exception of the baseline data (2.95 ± 0.24 vs. 3.38 ± 0.13, *p* = 0.107; 3.34 ± 0.22 vs. 4.01 ± 0.12, *p* = 0.010; and 3.28 ± 0.22 vs. 3.91 ± 0.13, *p* = 0.015, respectively).

### 3.4 Scatter plots showing BRH with blood glucose and HbA_1c_ levels

**FIGURE 3:**
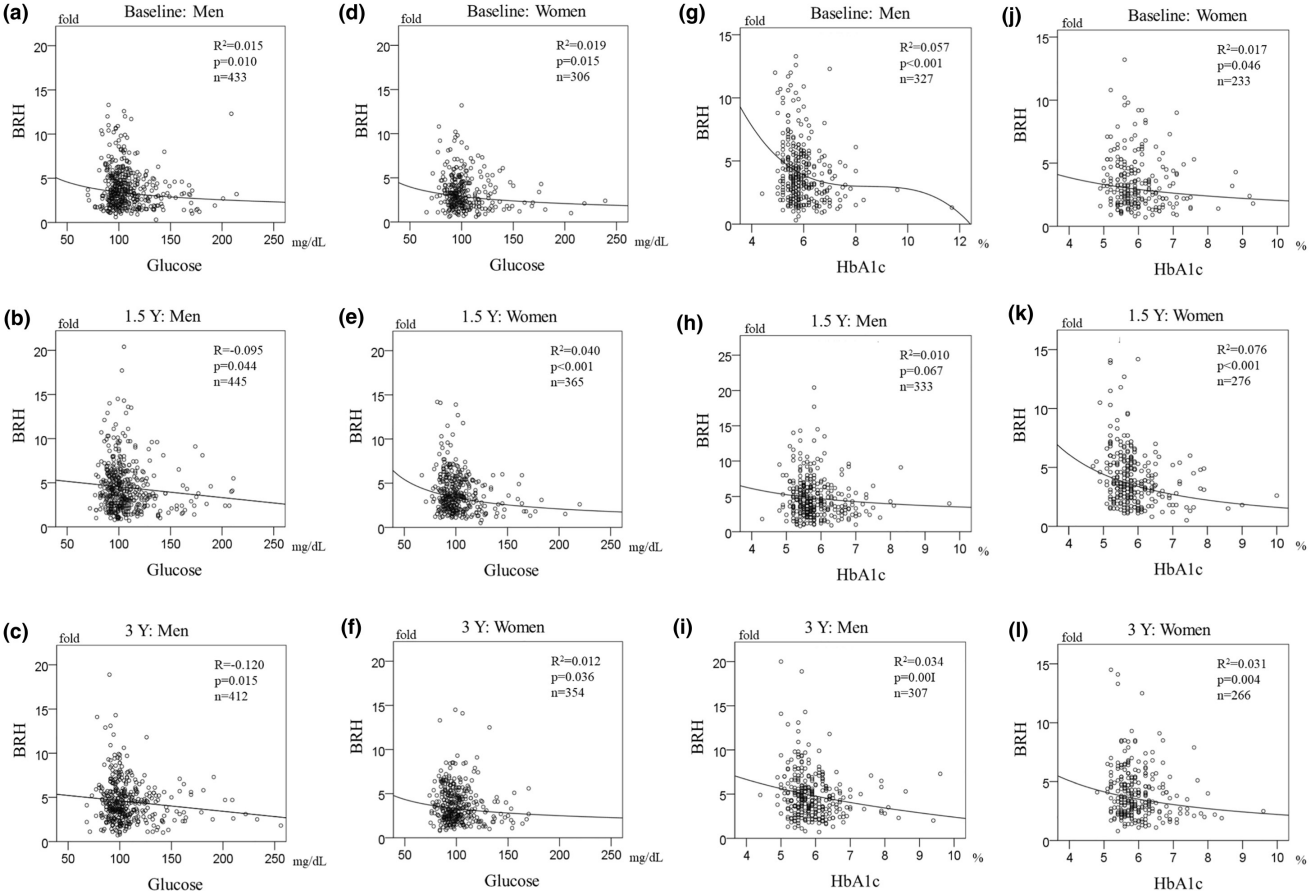
Scatter plots showing the relationship between BRH and blood glucose and glycated hemoglobin levels at each time point. The graphs show the correlation of BRH with blood glucose levels (a–f) and HbA_1c_ (g–l) at baseline, 1.5 years, and 3 years. The relationship is shown separately for men (a–c, g–i) and women (d–f, j–l). BRH, brachial reactive hyperemia; HbA_1c_, glycated hemoglobin; Y, years.

**FIGURE 4:**
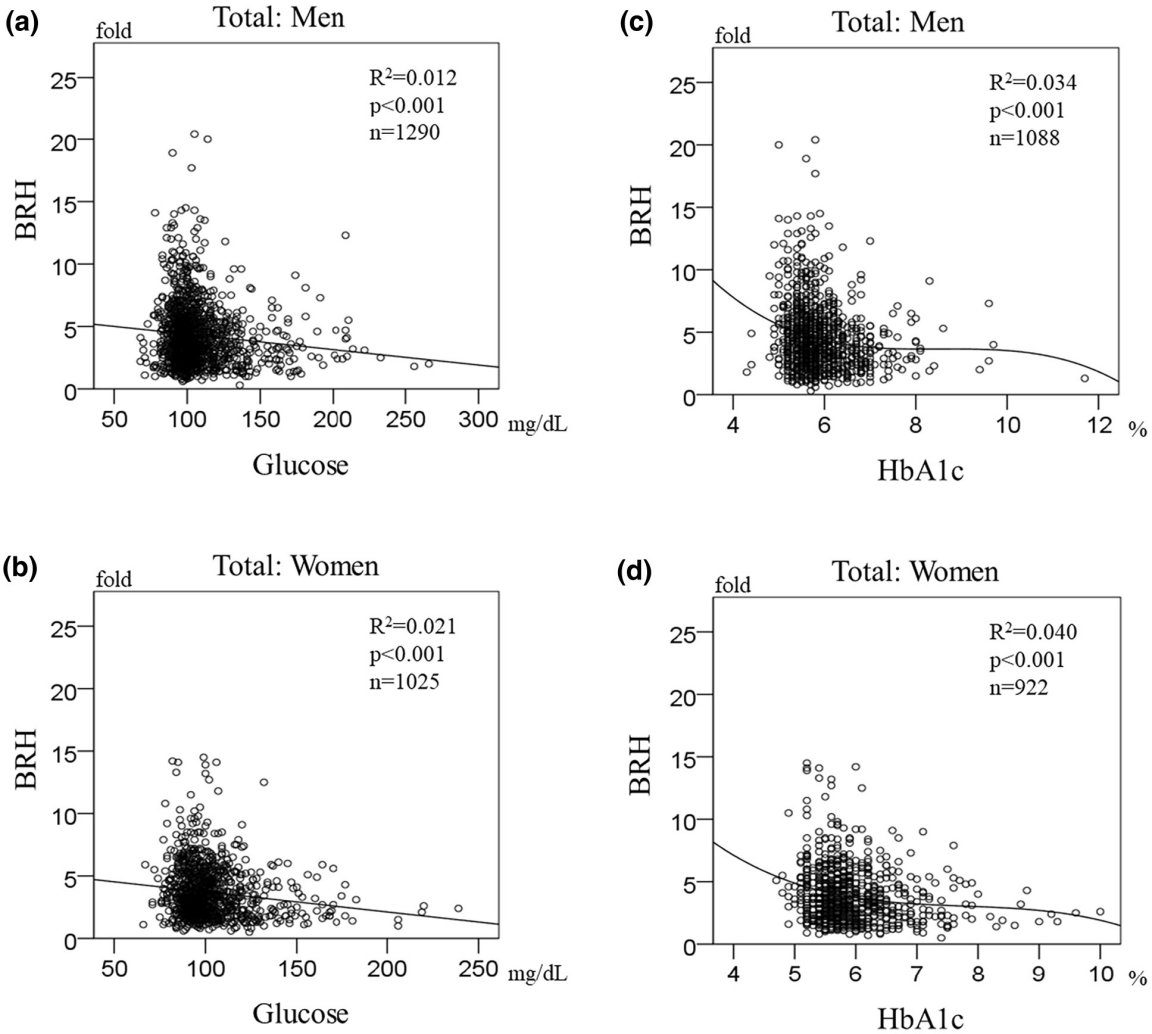
Scatter plots showing the measurements accumulated over 3 years. The graphs show the correlations of BRH with blood glucose levels in men (a) and women (b) and those of BRH with HbA_1c_ in men (c) and women (d). Few patients had high BRH when blood glucose or HbA_1c_ exceeded 126 mg/dL or 6.5%, (i.e., the diagnostic threshold for type 2 diabetes mellitus). BRH, brachial reactive hyperemia; HbA_1c_, glycated hemoglobin.

Figure 3 shows a scatter plot of BRH versus serum glucose and HbA_1c_ at each time point. BRH had a small negative correlation with blood glucose and HbA_1c_. BRH reached its peak when blood glucose was around 100 mg/dL and HbA_1c_ was below 6.0%. Of note was that a few subjects had high BRH when blood glucose was higher than 126 mg/dL or HbA_1c_ was above 6.5%, which is the diagnostic threshold for T2DM. This finding suggests that hyperglycemia has an effect on BRH. This trend is evident in the cumulative data (Figure 4).

### 3.5 Regression analysis to elucidate factors contributing to BRH

Table 3 shows the results of the univariate regression analyses performed to identify independent factors associated with BRH at each time point. The results of multivariate analyses with the stepwise model are also shown. In univariate regression analyses, T2DM had a negative association with BRH irrespective of sex except at baseline in women. BRH was negatively associated with BMI, pulse pressure, and positively associated with brachial artery diameter, diastolic blood pressure before examination. In multivariate analysis, T2DM was a significant independent predictor of BRH over time in men. BMI, brachial artery diameter, and diastolic blood pressure were cofactors although the composition varied at each time point. Daily alcohol consumption  was associated with increased BRH in women (Table 3).

**TABLE 3:** Univariate and multivariate regression analyses for BRH at each year.

A									
Men	Baseline	(*n* = 433)	*p* value	1.5 years	(*n* = 445)	*p* value	3 years	(*n* = 412)	*p* value
	β	t		β	t		β	t	
Age (years)	−0.102	−2.122	**0.034**	−0.188	−4.030	**<0.001**	−0.139	−2.846	**0.005**
BMI (kg/m^2^)	−0.159	−3.351	**0.001**	−0.120	−2.534	**0.012**	−0.081	−1.643	0.101
T2DM (yes, 1; no, 0)	−0.192	−4.067	**<0.001**	−0.162	−3.466	**0.001**	−0.158	−3.250	**0.001**
Hypertension (yes, 1; no, 0)	0.066	1.367	0.172	0.068	1.425	0.155	0.102	2.086	**0.038**
Hyperlipidemia (yes, 1; no, 0)	−0.157	−3.304	**0.001**	−0.114	−2.410	**0.016**	−0.099	−2.016	**0.044**
Alcohol consumption (yes, 1; no, 0)	0.093	1.941	0.053	0.068	1.437	0.151	0.086	1.745	0.082
Current smoking (yes, 1; no, 0)	−0.094	−1.966	**0.050**	−0.046	−0.963	0.336	−0.054	−1.087	0.278
Total cholesterol (mg/dL)	0.073	1.524	0.128	0.166	3.548	**<0.001**	0.145	2.969	**0.003**
Brachial artery diameter at baseline (mm)	0.112	2.342	**0.020**	0.052	1.105	0.270	0.065	1.315	0.189
Heart rate (bpm)	−0.164	−3.456	**0.001**	−0.040	−0.853	0.394	−0.040	−0.802	0.423
Diastolic BP (mm Hg)	0.166	3.487	**0.001**	0.158	3.358	**0.001**	0.238	4.971	**<0.001**
Pulse pressure (mm Hg)	−0.070	−1.465	0.144	−0.203	−4.358	**<0.001**	−0.162	−3.316	**0.001**
Glucose (mg/dL)	−0.114	−2.375	**0.018**	−0.095	−2.018	**0.044**	−0.120	−2.442	**0.015**
HbA_1c_ (%)^a^	−0.218	−4.035	**<0.001**	−0.099	−1.813	0.071	−0.172	−3.054	**0.002**

B									
Women	Baseline	(*n* = 306)	*p* value	1.5 years	(*n* = 365)	*p* value	3 years	(*n* = 354)	*p* value
	β	t		β	t		β	t	
Age (years)	−0.044	−0.764	0.445	−0.136	−2.616	**0.009**	−0.048	−0.908	0.365
BMI (kg/m^2^)	−0.154	−2.723	**0.007**	−0.157	−3.021	**0.003**	−0.129	−2.433	**0.015**
T2DM (yes, 1; no, 0)	−0.088	−1.546	0.123	−0.136	−2.620	**0.009**	−0.120	−2.273	**0.024**
Hypertension (yes, 1; no, 0)	0.108	1.896	0.059	0.072	1.374	0.170	0.042	0.781	0.435
Hyperlipidemia (yes, 1; no, 0)	−0.028	−0.495	0.621	−0.034	−0.646	0.519	−0.107	−2.015	**0.045**
Alcohol consumption (yes, 1; no, 0)	0.129	2.263	**0.024**	0.257	5.076	**<0.001**	0.218	4.196	**<0.001**
Regular exercise (yes, 1; no, 0)	0.103	1.805	0.072	−0.028	−0.532	0.595	−0.042	−0.785	0.433
Current smoking (yes, 1; no, 0)	−0.099	−1.731	0.084	0.080	1.528	0.127	0.040	0.759	0.448
Triglycerides (mg/dL)	−0.145	−2.548	**0.011**	−0.082	−1.572	0.117	−0.050	−0.945	0.345
Brachial artery diameter at baseline (mm)	0.108	1.889	0.060	0.146	2.804	**0.005**	0.075	1.416	0.158
Heart rate (bpm)	−0.148	−2.608	**0.010**	−0.059	−1.119	0.264	−0.072	−1.356	0.176
Systolic BP (mm Hg)	−0.135	−2.380	**0.018**	−0.033	−0.621	0.535	0.031	0.582	0.561
Diastolic BP (mm Hg)	0.088	1.539	0.125	0.196	3.812	**<0.001**	0.159	3.017	**0.003**
Pulse pressure (mm Hg)	−0.200	−3.562	**<0.001**	−0.182	−3.520	**<0.001**	−0.087	−1.632	0.104
Glucose (mg/dL)	−0.138	−2.428	**0.016**	−0.185	−3.594	**<0.001**	−0.096	−1.812	0.071
HbA_1c_ (%)^b^	−0.121	−1.853	0.065	−0.252	−4.305	**<0.001**	−0.167	−2.759	**0.006**

C									
Men	Baseline	(*n* = 433)	*p*‐value	1.5 years	(*n* = 445)	*p*‐value	3 years	(*n* = 412)	*p*‐value
	β	t		β	t		β	t	
Age (years)	−0.106	−2.188	**0.029**	−0.147	−2.841	**0.005**	−0.062	−1.171	0.242
BMI (kg/m^2^)	−0.204	−4.278	**<0.001**	−0.159	−3.321	**0.001**	−0.114	−2.277	**0.023**
T2DM (yes, 1; no, 0)	−0.102	−2.179	**0.030**	−0.102	−2.199	**0.028**	−0.097	−1.987	**0.048**
Current smoking (yes, 1; no, 0)	−0.089	−1.977	**0.049**						
Brachial artery diameter at baseline (mm)	0.120	2.623	**0.009**						
Heart rate (bpm)	−0.187	−4.042	**<0.001**						
Diastolic BP (mm Hg)	0.189	3.950	**<0.001**	0.119	2.479	**0.014**	0.220	4.432	**<0.001**
Pulse pressure (mm Hg)				−0.118	−2.421	**0.016**	−0.107	−2.148	**0.032**

D.									
Women	Baseline	(*n* = 306)	*p* value	1.5 years	(*n* = 365)	*p* value	3 years	(*n* = 354)	*p* value
	β	t		β	t		β	t	
Age (years)	0.012	0.183	0.855	−0.090	−1.712	0.088	−0.005	−0.088	0.930
BMI (kg/m^2^)	−0.169	−2.985	**0.003**	−0.154	−2.927	**0.004**	−0.109	−2.030	**0.043**
Alcohol consumption (yes, 1; no, 0)	0.166	2.734	**0.007**	0.192	3.751	**<0.001**	0.184	3.333	**0.001**
Current smoking (yes, 1; no, 0)	−0.165	−2.755	**0.006**						
Brachial artery diameter at baseline (mm)				0.214	4.305	**<0.001**			
Heart rate (bpm)	−0.176	−3.010	**0.003**						
Diastolic BP (mm Hg)				0.158	3.225	**0.001**	0.105	1.956	0.051
Pulse pressure (mm Hg)	−0.212	−3.390	**0.001**						
Glucose (mg/dL)				−0.152	−3.086	**0.002**			

Abbreviations: BMI, body mass index; BP, blood pressure; T2DM, type 2 diabetes mellitus.

*Note*: A, B: univariate regression model, C, D: multivariate stepwise regression model. Values in (A,C) are for men and those in (B, D) are for women. Each analysis was independently performed at baseline, 1.5 years, and 3 years. The analyses used independent variables comprising laboratory data, physiological measurements, and clinical information, which were taken at each assessment time. In the multivariate stepwise regression model, independent factors were selected with *p* < 0.1 for each year. The number of subjects used for the regression models are indicated in parentheses. The adjusted R^2^ values for the stepwise models at baseline, 1.5 years, and 3 years were 0.132, 0.096, and 0.092, respectively, in men (C) and 0.107, 0.162, and 0.071 in women (D). *p* values <0.05 are shown in bold.

^a^ *n* = 327, *n* = 333, and *n* = 307 for baseline, 1.5 years, and 3 years, respectively.

^b^ *n* = 233, *n* = 276, and *n* = 266 for baseline, 1.5 years, and 3 years, respectively.

## 4 DISCUSSION

This study demonstrated that BRH is attenuated in individuals with T2DM. The strength of the study is that it eliminated errors attributed to differences in test procedures by standardizing the protocol between institutions. We used the same device for measurement of BRH, and the methodology allowed us to obtain consistent results in repeated measurements. To our knowledge, this study is the first to demonstrate a relationship between T2DM and reactive hyperemia in the brachial arteries by means of large data sets using a semiautomatic measurement system for FMD.

An early study failed to demonstrate a difference in BRH between patients with and without T2DM, possibly because of small sample size (Spallarossa et al., 2001). However, subsequent studies suggested that hyperemic blood flow was reduced in patients with diabetes or prediabetes. A Framingham Heart Study of 2045 subjects found that diastolic shear stress during reactive hyperemia was negatively associated with age, BMI, pulse pressure, and fasting glucose levels (Mitchell et al., 2004). These same factors were negatively associated with BRH in our present study. Obesity reduces production of nitric oxide (NO) via insulin resistance (Engin, 2017). Hyperglycemia is accompanied by insulin resistance and high blood viscosity, which increases shear stress on the vessel wall (Irace et al., 2014). Plasma glucose levels are negatively associated with blood flow in the forearm during reactive hyperemia measured by mercury‐in‐rubber strain gauge plethysmography (Keymel et al., 2011). Even in the short term, physical inactivity causes insulin resistance and decreases hyperemic flow in the brachial artery in both arms and in the leg arteries (Hamburg et al., 2007). Our present findings are in line with those previously reported and have further characterized the relationship between BRH and hyperglycemia. BRH was suppressed in subjects with hyperglycemia but was not increased in some with normoglycemia owing to the influence of other factors. This relationship may explain why the attenuated hyperemic response in the brachial artery in patients with diabetes has received little attention.

The mechanisms of reactive hyperemia have been investigated. Factors contributing to reactive hyperemia include endothelium‐mediated NO synthesis, endogenous adenosine (Carlsson et al., 1987; Costa et al., 1999; Meijer et al., 2008), potassium channel functions (Crecelius et al., 2013), and prostaglandins (Carlsson et al., 1987; Engelke et al., 1996; Kilbom & Wennmalm, 1976; Taylor et al., 2014). These factors promote hyperpolarization of vascular smooth muscle cells, resulting in vasorelaxation (Edwards et al., 2010; Félétou, 2009). Hyperemic blood flow is assessed in two phases: peak and postpeak. There have been reports suggesting that reactive hyperemia is attenuated in the presence of the NO synthase inhibitor NG‐monomethyl‐L‐arginine (Higashi et al., 2001; Meredith et al., 1996). However, NO was found to be less involved in peak blood flow measured by strain gauge plethysmography with a venous occlusion technique (Crecelius et al., 2013; Tagawa et al., 1994), suggesting that the initial response is not endothelium‐dependent. In this regard, BRH takes peak velocities into consideration and might reflect factors other than production of and signaling by NO.

Peak blood flow in the forearm is primarily determined by activation of inwardly rectifying potassium channels (K_IR_) though the total amount of BRH is determined by both K_IR_ and Na+/K + ‐ATPase (Crecelius et al., 2013). Obesity‐induced endothelial dysfunction was found to be mediated by a loss of flow sensitivity in the K_IR_ channel in both mouse and human mesenteric arteries (Fancher et al., 2020). Glibenclamide, an antidiabetic drug, inhibits ATP‐dependent potassium (K_ATP_) channels and attenuates reactive and functional hyperemia (Banitt et al., 1996; Bijlstra et al., 1996), although there are arguments to the contrary (Spallarossa et al., 2001; Farouque & Meredith, 2003). K_ATP_ channels are thought to contribute to delivery of oxygen in skeletal muscle during exercise, and blockade of K_ATP_ channels with glibenclamide attenuates exercise‐induced hyperemia in rats (Holdsworth et al., 2015). Thus, treatment with glibenclamide may worsen vascular function. Although the antidiabetic drugs used by our study participants were not investigated in detail, the overall low antidiabetic medication rate (36%) suggests that use of glibenclamide would have been low. Therefore, our results cannot be attributed to drug therapy alone. The effects of T2DM on the expression and functioning of potassium and calcium channels has been studied in vascular smooth muscle cells (Nieves‐Cintrón et al., 2021; Pereira da Silva et al., 2022). However, functional changes in endothelial ion channels in diabetic patients are less understood owing to the difficulty of isolating native endothelial cells (Welsh et al., 2018).

The results of our study also indicate that there is a sex difference in the factors contributing to BRH. BRH was higher among habitual consumers of alcohol, particularly if they were female. The relationship between alcohol consumption and hyperemic blood flow in the brachial artery has not been well studied and was not found in a previous study with a small sample size (Teragawa et al., 2002). In terms of other tests of endothelial function, the effect of alcohol on FMD has been investigated extensively but whether it is sex‐related remains uncertain (Hwang et al., 2021). A previous FMD‐J study reported that light to moderate alcohol intake was not associated with FMD but that heavy alcohol intake was associated with deterioration of FMD in women (Oda et al., 2020). An association between heavy alcohol intake and worsening FMD has also been reported in men with coronary artery disease (Tanaka et al., 2016). Another study that included peripheral arterial tonometry showed that the reactive hyperemia index increased with red wine consumption (Cioni et al., 2013). Whether the increase in blood flow velocity caused by alcohol is beneficial to health must await future studies.

Our findings suggest that prevention of T2DM and glycemic control are important for maintenance of the arterial and arteriolar circulation. A decrease in reactive hyperemic velocity may be a type of circulatory disturbance that hastens the progression of arteriosclerosis at specific anatomical locations, such as the coronary arteries and arteries in the lower extremities. Ameliorating reactive hyperemia is important because the magnitude of shear stress on the surface of the vascular endothelium plays a major role in vasodilation (Koller & Bagi, 2002). Further research is needed to inform guidance regarding lifestyle and pharmacotherapy for maintenance of adequate reactive hyperemia in both sexes.

This study has several limitations. First, it evaluated the rate of increase in peak blood flow velocity. The brachial artery diameter is known to be larger in patients with risk factors for atherosclerosis (Maruhashi et al., 2018). A larger vessel diameter may result in attenuated peak hyperemia without a decrease in total blood flow after cuff release. However, previous studies in patients with peripheral arterial disease have reported that reactive hyperemic velocity, rather than hyperemic flow, is useful in predicting cardiovascular events (Huang et al., 2007), and the effect of T2DM on hyperemic blood flow and the area under the curve for shear rate requires further investigation. Second, the T2DM group in this study included individuals who were prediabetic at the time of enrollment and subsequently diagnosed with T2DM. It needs to be confirmed whether the same results can be obtained in patients with T2DM and more advanced atherosclerosis, such as coronary artery disease. Third, both the T2DM and non‐T2DM groups had other cardiovascular risk factors such as hypertension and hyperlipidemia. These conditions generally progress simultaneously. Furthermore, the timing of treatment for hypertension in patients with T2DM differs from that in those without T2DM (Whelton et al., 2022). We therefore performed binary classification according to T2DM status in patients with similar backgrounds. Fourth, the antidiabetic drugs used in the patients with T2DM is not known, other than metformin, sulfonylureas, pioglitazone, and dipeptidyl peptidase‐4 inhibitors were commonly used at the time of enrolment.

In conclusion, repeated measurement of BRH revealed that T2DM was associated with attenuated postocclusive reactive hyperemia of the brachial artery.

## AUTHOR CONTRIBUTIONS

H.T., T.K., T.S., T.I., S.U., T.Y., T.F., K.K., T.I., S.K., Y.T., T.H., M.S., Y.I., K.N., K.M., Y O., T F., H.I., Y.H., A.Y., and B.T. conceived and designed research; B.T. performed experiments; N.M. analyzed data; N.M. interpreted results of experiments; N.M. prepared figures; N.M. drafted manuscript; B.T. and N.M. edited and revised manuscript; T.A. and B.T. approved final version of manuscript.

## FUNDING INFORMATION

This study was supported, in part, by a Grant‐in‐Aid from the Japanese. Atherosclerosis Prevention Fund. The work was supported by a Grant‐in‐aid for Scientific Research from the Ministry of Education, Culture, Sports, Science and Technology of Japan (20 K08503 to Masaki N).

## CONFLICT OF INTEREST STATEMENT

S. Koba received contract research funds from Denka Co. Ltd. Y. Higashi received consulting fees related to this study from Mitsubishi Tanabe, as well as honoraria and grants from Teijin, Boehringer Ingelheim, MSD, Sanofi, AstraZeneca, Kyowa Hakko Kirin, Takeda, Astellas, Daiichi Sankyo, Mochida, Nihon Kohden, Shionogi, and Nippon. Sigmax, Sanwa Kagaku Kenkyusho, Unex, and Kao; and honoraria from Radiometer, Omron, Sumitomo Dainippon, Otsuka, Torii, Kowa, Fujiyakuhin, Amgen, Nippon Shinyaku, Itamar, Bayer, Eli Lilly, and Ono.
